# Supplementary material for: Indels allow antiviral proteins to evolve functional novelty inaccessible by missense mutations
Source: Cell Genom. 2025 Mar 25;5(6):100818. doi: 10.1016/j.xgen.2025.100818 (PMC12230231; doi:10.1016/j.xgen.2025.100818)
Supplement: Document S1. Figures S1–S5 [file mmc1.pdf]

Cell Genomics, Volume *5*

Supplemental information

Indels allow antiviral proteins to evolve functional novelty inaccessible by missense mutations

Jeannette L. Tenthorey, Serena del Banco, Ishrak Ramzan, Hayley Klingenberg, Chang Liu, Michael Emerman, and Harmit S. Malik

Supplement for:

"Indels allow antiviral proteins to evolve functional novelty inaccessible by missense mutations"

Jeannette L. Tenthorey, Serena del Banco, Ishrak Ramzan, Hayley Klingenberg, Chang Liu, Michael Emerman, Harmit S. Malik

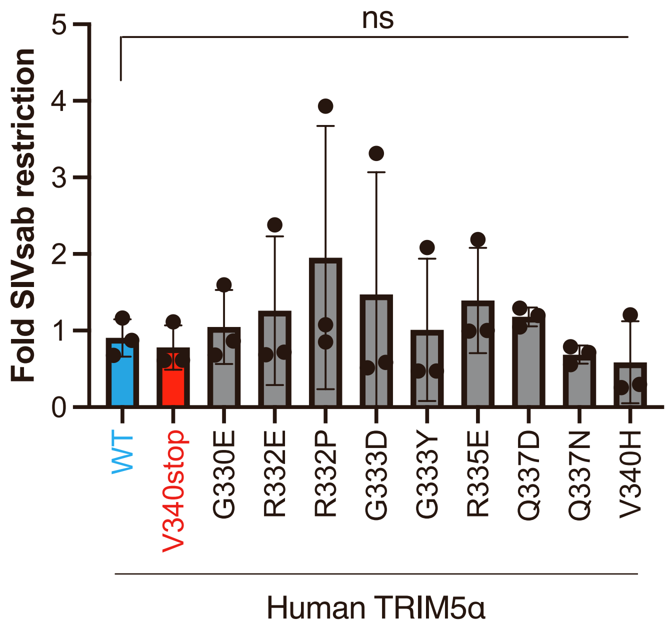

Figure S1. Validation of human TRIM5α deep mutational scanning results, related to Figure 1. Single missense variants of human TRIM5α (which were not enriched in the gain-of-SIVsab restriction screen in Figure 1C) were stably expressed in CRFK cells and challenged with SIVsab; fold restriction was calculated as an increase in infectious dose (ID_5) compared to cells expressing empty vector (n = 3 independent experimental measurements for each cell line). Bars, mean; error bars, SD; ns, not significant; Kruskal-Wallis one-way ANOVA with Dunn's multiple comparison (vs. WT) correction. See Data S1 for source data.

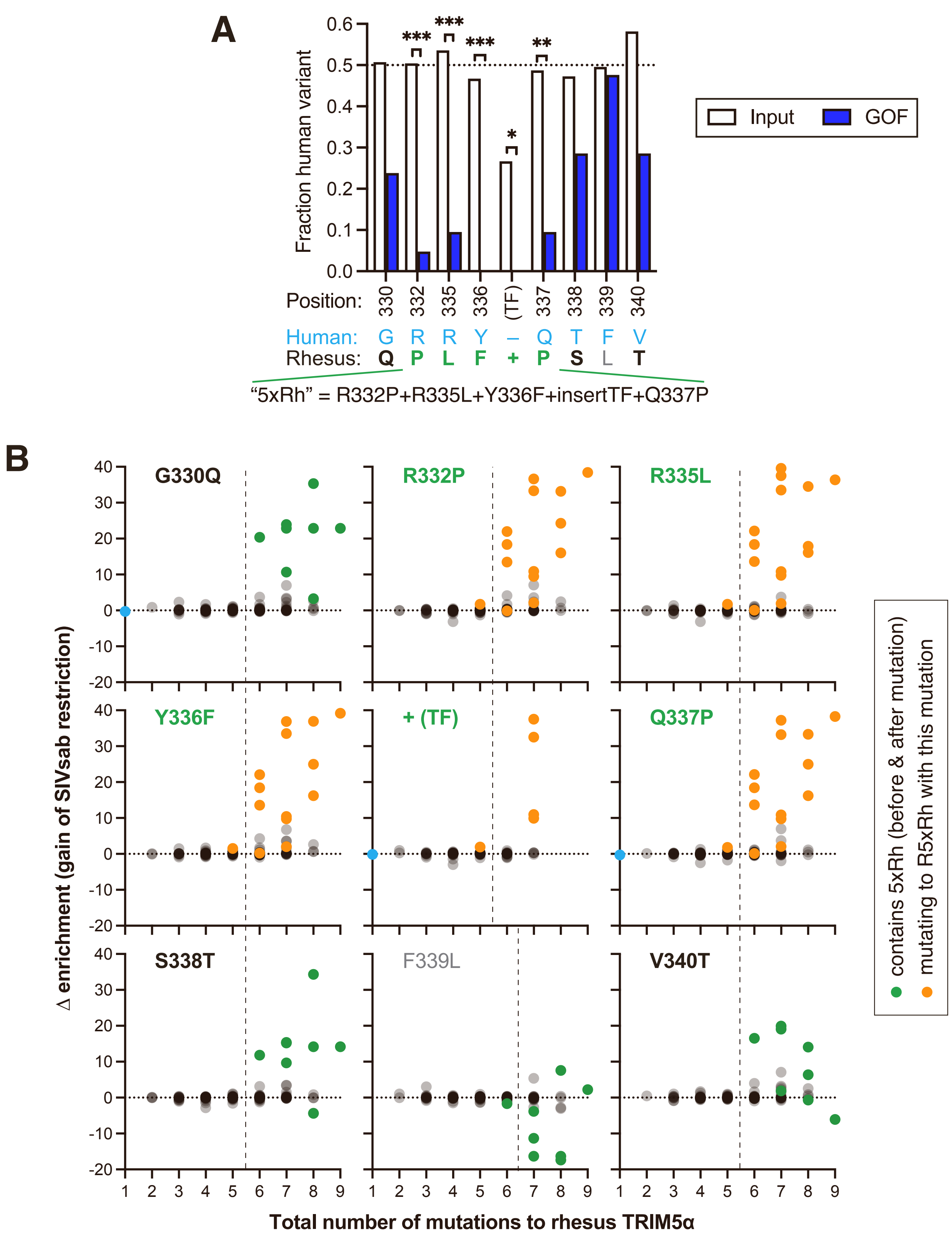

Figure S2. No rhesus-like mutation confers SIVsab restriction onto human TRIM5α in the absence of at least 5 other mutations, related to Figure 2. Results from the human/rhesus

combinatorial v1 library screen for human TRIM5α gain of SIVsab restriction (Figure 2), averaged across 2 independent experimental replicates. (**A**) The fraction of variants encoding the human sequence at each position for the input library (all variants detected in unsorted cells) and gain-of-function (GOF) variants (defined as outscoring all frameshift variants [see Materials and Methods for frameshift origin] in both replicates). The 5 statistically required rhesus mutations (5xRh) are highlighted in green; other beneficial rhesus mutations (positions that display a modest preference against the human variant) are bolded in black. *$p < 0.05$; **$p < 0.01$; ***$p < 0.001$; Chi-square test. (**B**) The change in enrichment (in SIVsab restrictor pool, averaged across 2 replicates) as human TRIM5α variants acquire a required (green), beneficial (black), or other (gray) rhesus TRIM5α mutation was calculated by subtracting the average enrichment for each variant lacking the mutation from the matched variant that acquired the mutation but was otherwise identical in sequence. Variants are plotted by the total number of rhesus TRIM5α mutations (after acquiring the indicated mutation). Matched pairs are colored by WT starting variant (cyan), variant matched pairs that both contain the 5xRh required mutations (green), and variants that acquire all 5xRh mutations after they acquire the indicated mutation (orange). The dashed line at y = 0 indicates neutral mutations with no effect on SIVsab restriction. The dashed line at x = 5.5 highlights the requirement for 5 pre-existing mutations (6 total upon acquisition of the indicated mutation) to improve SIVsab restriction (Δ enrichment > 0) in all backgrounds. The F339L mutation does not contribute to restriction, and a 7th mutation is required for F339L variants. See Data S2 for source data.

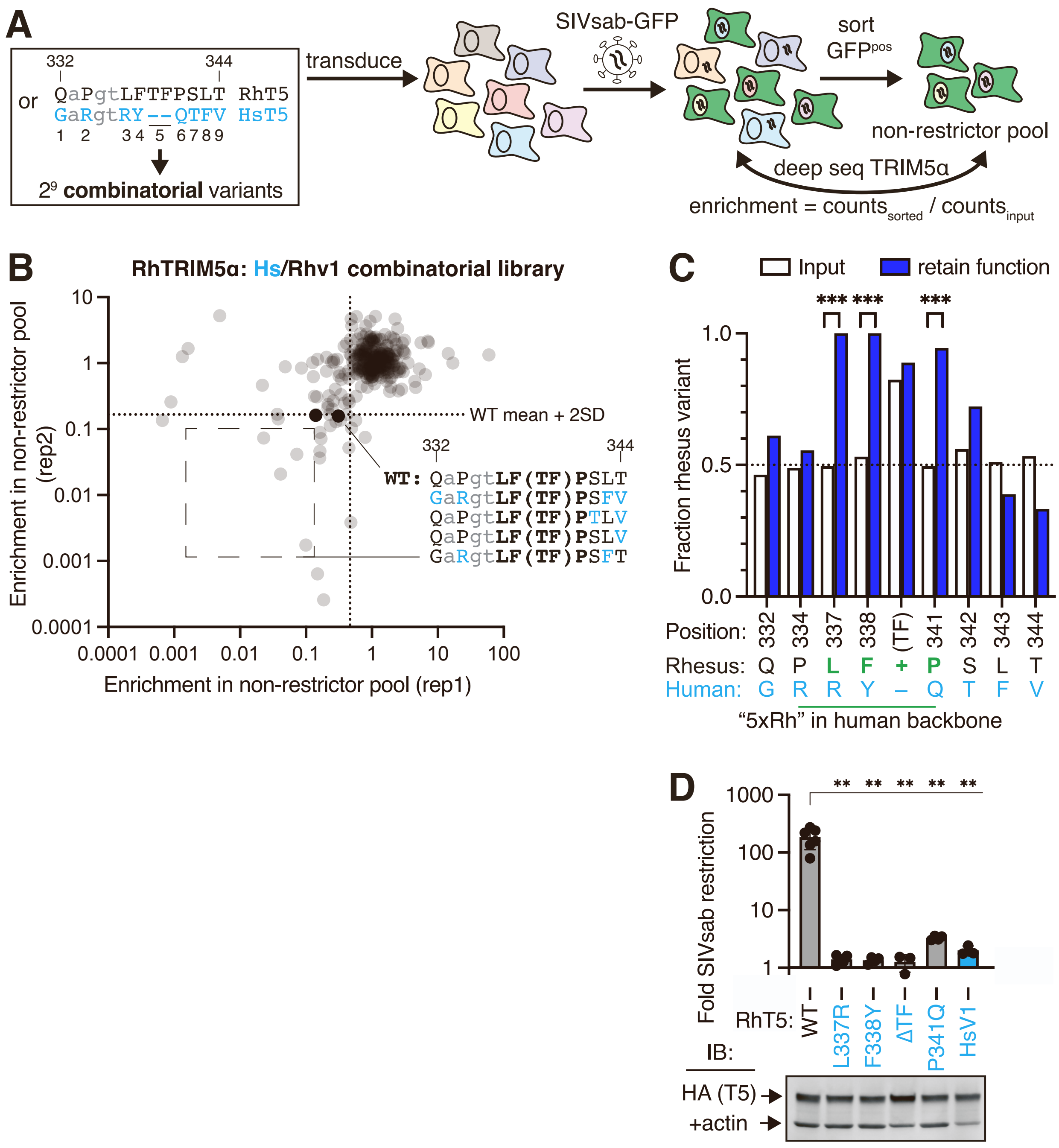

Figure S3. Rhesus TRIM5α shares the requirement for rhesus-like sequence at 4 positions (337-341) of the 5 required by human TRIM5α, related to Figure 2. (**A**) A rhesus TRIM5α library combinatorially sampling the human or rhesus variant at each v1 position was expressed in CRFK cells, challenged with SIVsab-GFP, and sorted for GFP^{pos} cells to identify loss-of-function variants. (**B**) Most combinatorial variants (80%) are significantly enriched (> [WT (black) mean + 2 SD] in 2 independent replicates), decreasing the sensitivity of sequence preference analysis; subsequent analysis therefore focuses on the non-enriched, retention-of-function variants. The sequence of best-performing variants (dashed box) is colored by human (cyan) or rhesus (black) variant at each site. (**C**) The fraction of variants encoding the rhesus

TRIM5α sequence at each position for the input library (all variants detected in unsorted cells) and retention-of-function pools (defined as < [WT mean + 2 SD] in both replicates) highlights a requirement for the rhesus TRIM5α variant at 4 central v1 loop positions; these are a subset of the 5 rhesus-like mutations strictly required by human TRIM5α. Variants lacking the (TF) insertion were under-represented in the input library, reducing statistical power, but the requirement for the insertion was confirmed by mutagenesis in (D). (**D**) Rhesus TRIM5α variants were individually expressed in CRFK cells and challenged with SIVsab (n = 3 [ΔTF, HsV1], 4 [L337R, F338Y, P341Q] or 6 [WT] independent experiments) to confirm screen results: rhesus variants at positions 337-341 are all strictly required for SIVsab restriction. Stable expression of all TRIM5α-HA constructs in CRFK cells was confirmed by immunoblot against HA. Bars, mean; error bars, SD. ns, not significant; *$p < 0.05$; ***$p < 0.001$; (C) Chi-square test; (D) Welch one-way ANOVA with Dunnett's T3 multiple comparisons correction (vs. WT). See Data S3 for source data.

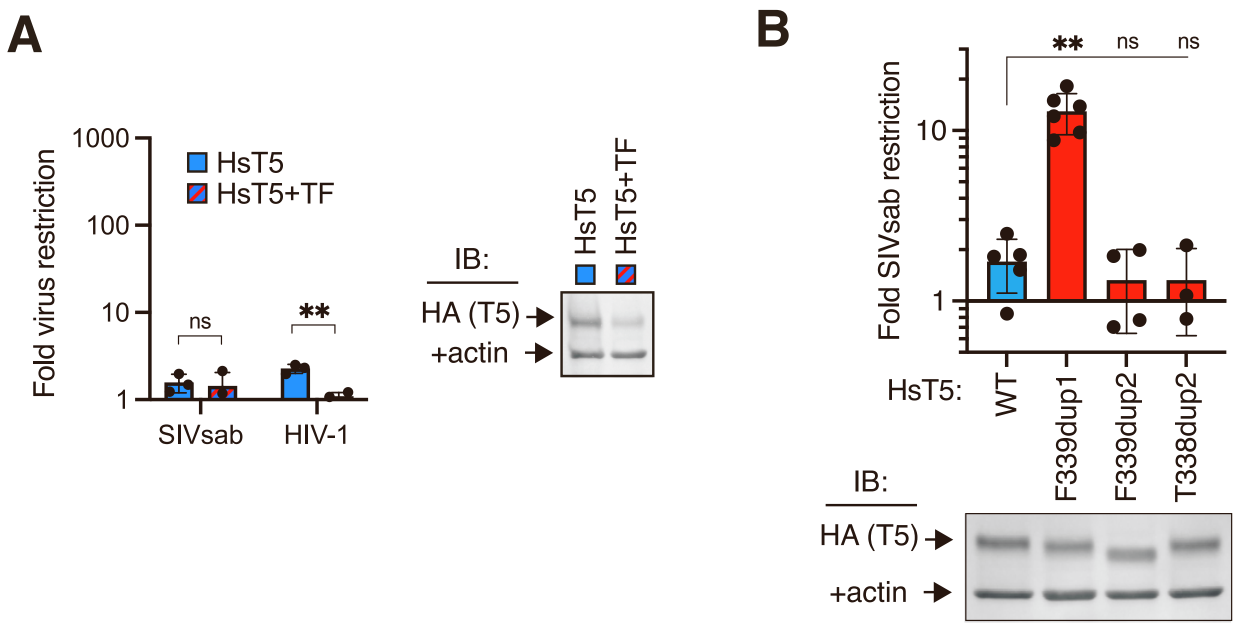

Figure S4. The effects of insertion mutations are context-dependent, related to Figure 4. (**A**) A rhesus-like insertion is insufficient to confer human TRIM5α with antiviral function. Human TRIM5α containing the rhesus TF339-340 insertion at the orthologous position (before Q337) was expressed in CRFK cells and challenged with the indicated viruses (n = 3 independent experiments). TRIM5α expression levels were analyzed by immunoblot (IB) against the C-terminal HA tag. The drop in anti-HIV-1 potency may be explained by decreased expression or stability of human TRIM5α containing the rhesus TRIM5α insertion. (**B**) Duplication of an additional residue before or after F339 (yielding amino acid sequence FVFV or TFTF) does not allow human TRIM5α to inhibit SIVsab when expressed in CRFK cells (n = 3 [T338dup2], 4 [F339dup2], 5 [WT] or 6 [F339dup1] independent experiments). Antiviral function does not correlate with TRIM5α expression levels. Bars, mean; error bars, SD. ns, not significant; ***p* < 0.01; (A) student's unpaired 2-tailed t test; (B) Welch one-way ANOVA with Dunnett's T3 multiple comparisons test (vs. WT). See Data S4 for source data.

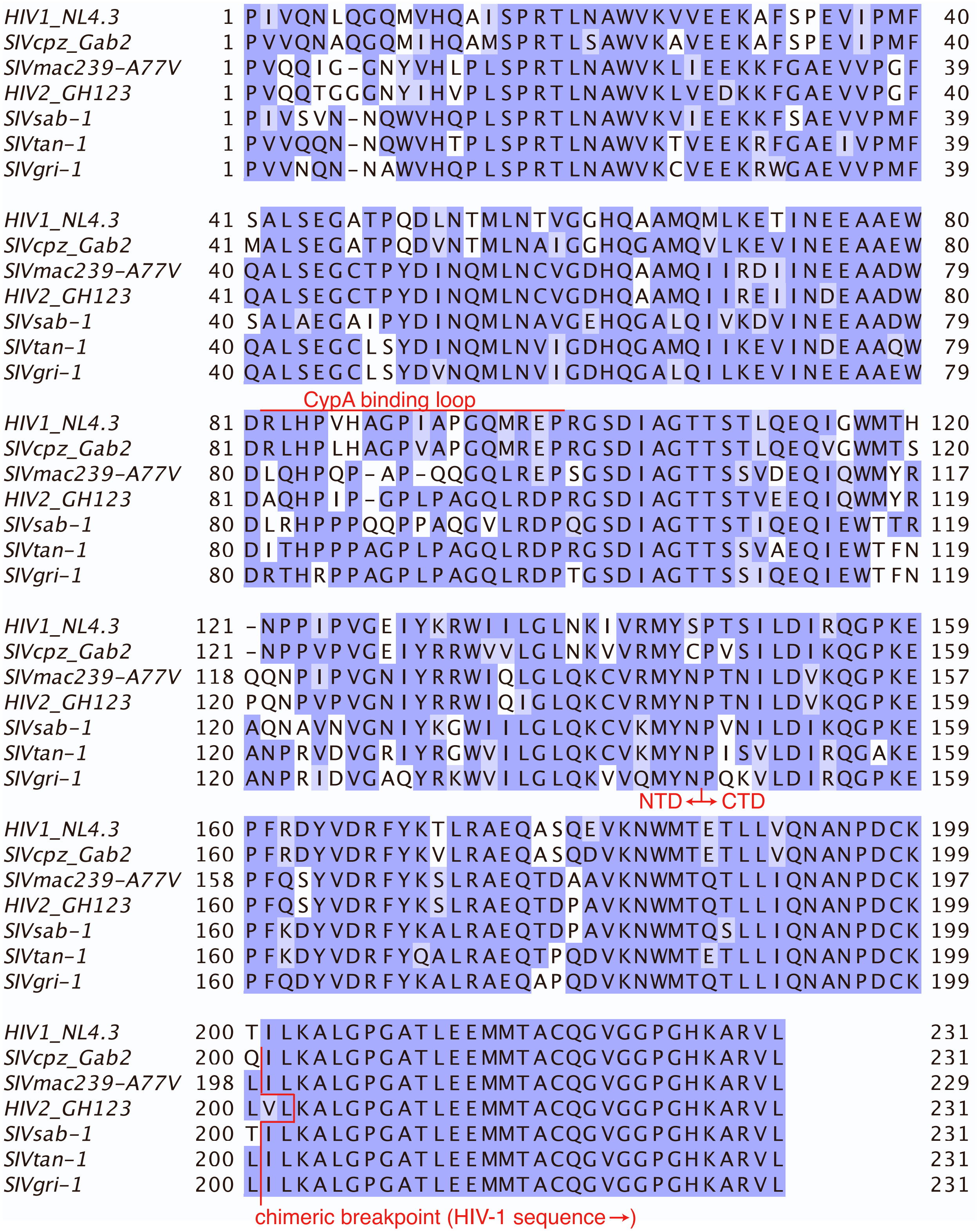

Figure S5. Lentiviral capsid sequences used in this study, related to STAR Methods. All capsids were chimerized with C-terminal HIV-1 sequence at the indicated breakpoint and were expressed within the HIV-1 (NL4-3 strain) gag/pol to generate virus. The cyclophilin A (CypA)-binding loop, which has been implicated in TRIM5α recognition[S1,S2], and the domain boundary

between the N-terminal domain (NTD) and C-terminal domain (CTD) are indicated. Residue conservation is colored by BLOSUM62 score.

Supplemental References

S1. Kim, K., Dauphin, A., Komurlu, S., McCauley, S.M., Yurkovetskiy, L., Carbone, C., Diehl, W.E., Strambio-De-Castillia, C., Campbell, E.M., and Luban, J. (2019). Cyclophilin A protects HIV-1 from restriction by human TRIM5α. Nature Microbiology *4*, 1–17. https://doi.org/10.1038/s41564-019-0592-5.

S2. Selyutina, A., Persaud, M., Simons, L.M., Bulnes-Ramos, A., Buffone, C., Martinez-Lopez, A., Scoca, V., Nunzio, F.D., Hiatt, J., Marson, A., et al. (2020). Cyclophilin A Prevents HIV-1 Restriction in Lymphocytes by Blocking Human TRIM5α Binding to the Viral Core. Cell Reports *30*, 3766-3777.e6. https://doi.org/10.1016/j.celrep.2020.02.100.
