# Supplementary material for: Indels allow antiviral proteins to evolve functional novelty inaccessible by missense mutations
Source: Cell Genom. 2025 Mar 25;5(6):100818. doi: 10.1016/j.xgen.2025.100818 (PMC12230231; doi:10.1016/j.xgen.2025.100818)
Supplement: Data S8. Raw images of TRIM5α immunoblot analysis when stably expressed in CRFK cells, related to STAR Methods [file mmc11.pdf]

NB: all blots simultaneously probed for:

- Primary Ab: rabbit anti-HA (large band ~56kDa) and rabbit anti-actin (lower band, 42kDa)
- Secondary Ab: anti-rabbit 800
- Imaged for both colors on Licor imager

Figure 1D: Immunoblot of HsT5 single missense mutants.

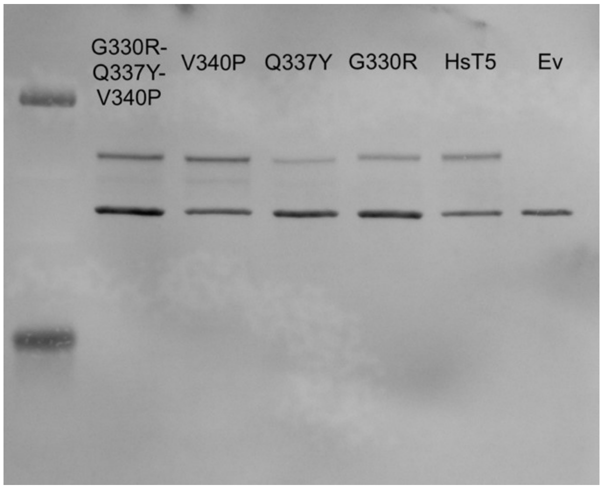

Samples from 231218; File name: 20231220-Gel2-label or 20231220-Gel2-[IRlong].tif

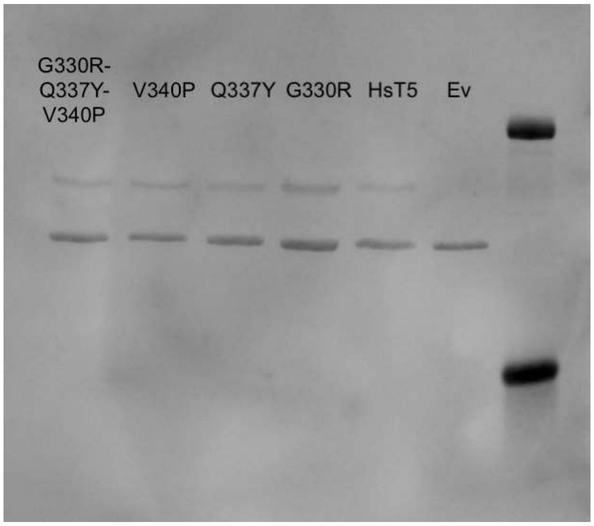

Samples from 231215 run on 240103; File name: 240103-WB1-3&29-[IRLong].tif

Samples from 231215- first run on 231220
Taken from files named 20231220-Gel2-[IRlong].tif and 20231220-Gel1-[IRlong].tif

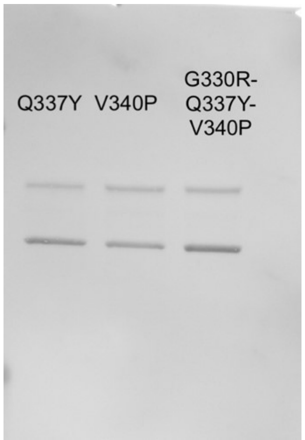

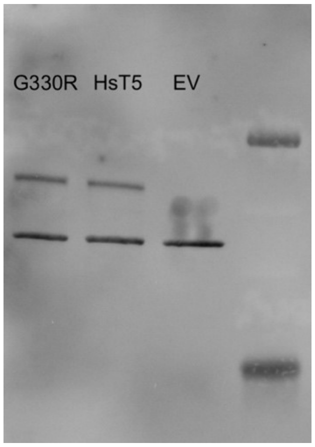

Figure 2E: Immunoblot of HsT5 combinatorial mutants

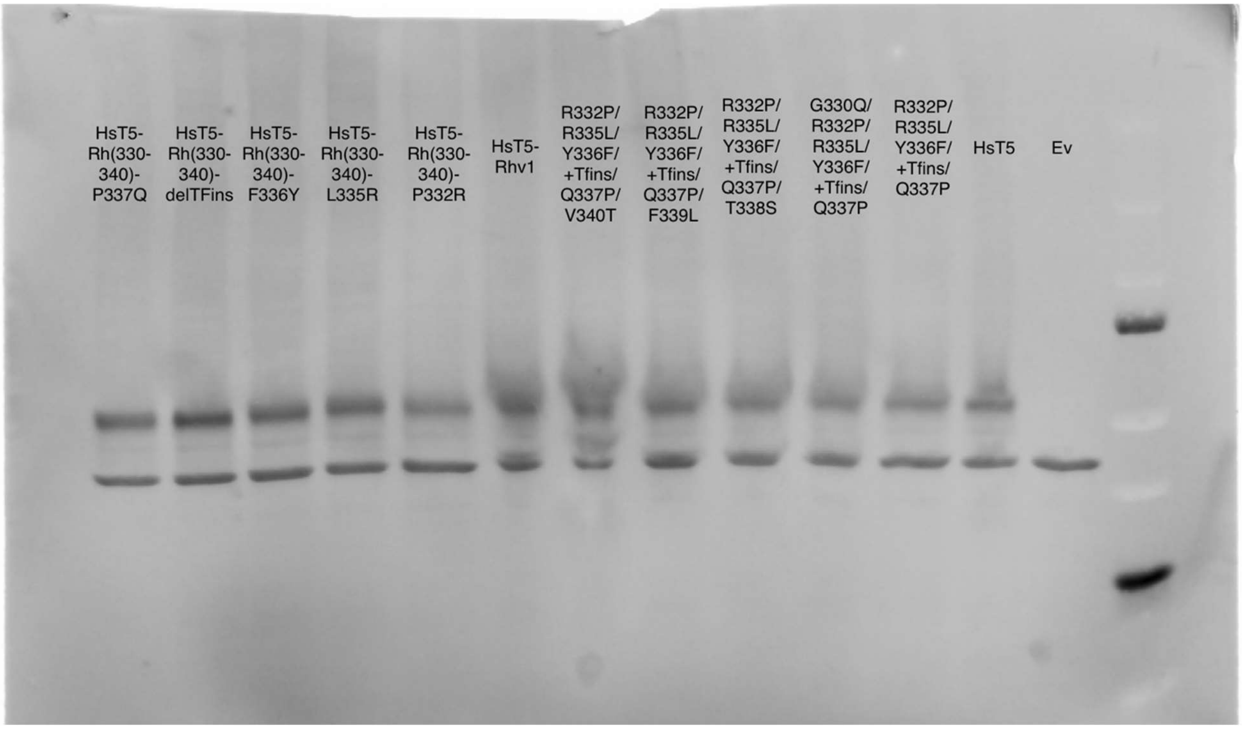

Samples collected on 240104; File name: 20240109-WB9-13&4-8-1-[IRlong].tif

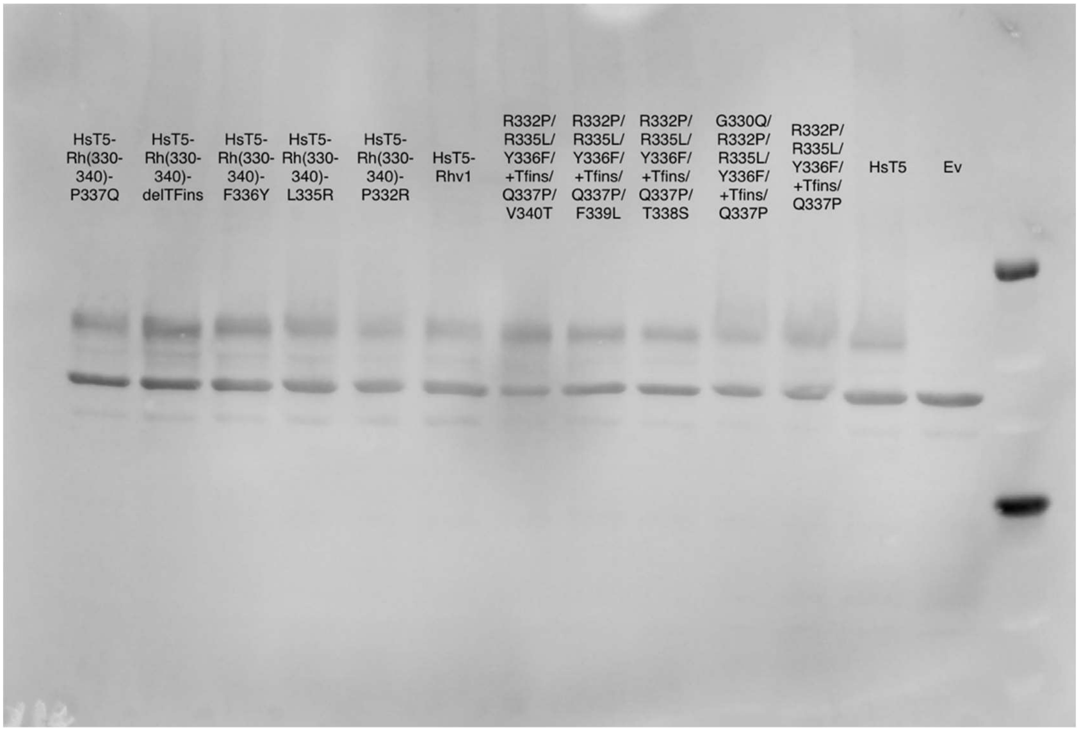

Samples collected on 240108; File name: 20240110-WB-9-13&4-8-2-[IRlong].tif

Figure 4C: immunoblot of HsT5 SIVsab gain-of-function indels

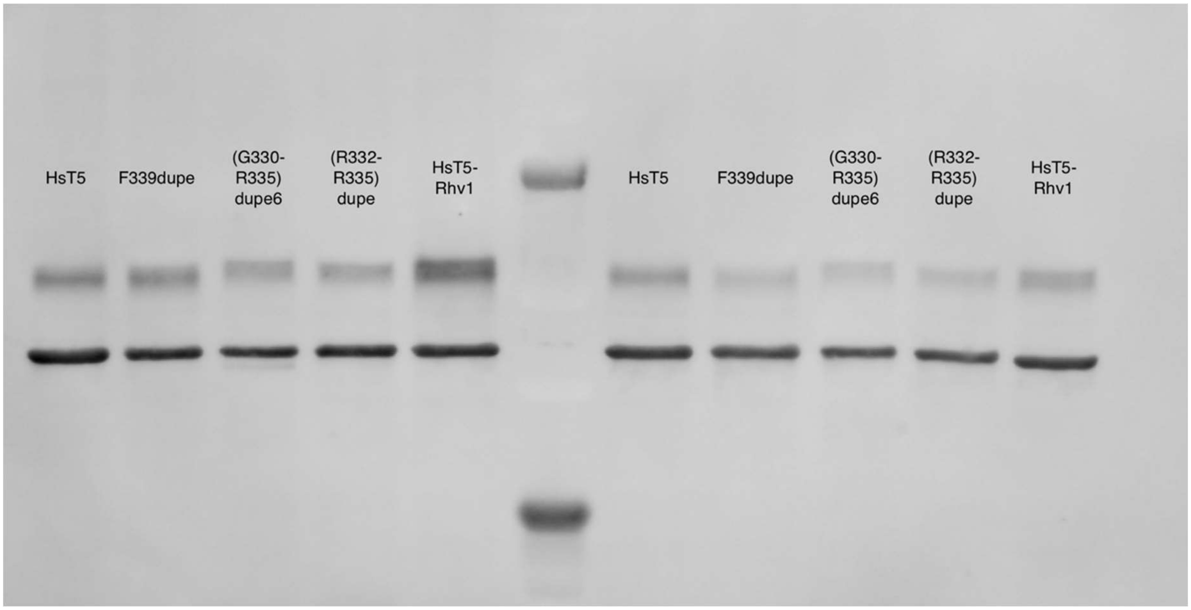

File name: 20240118-F339dupe&rhv1-[IRlong].tif

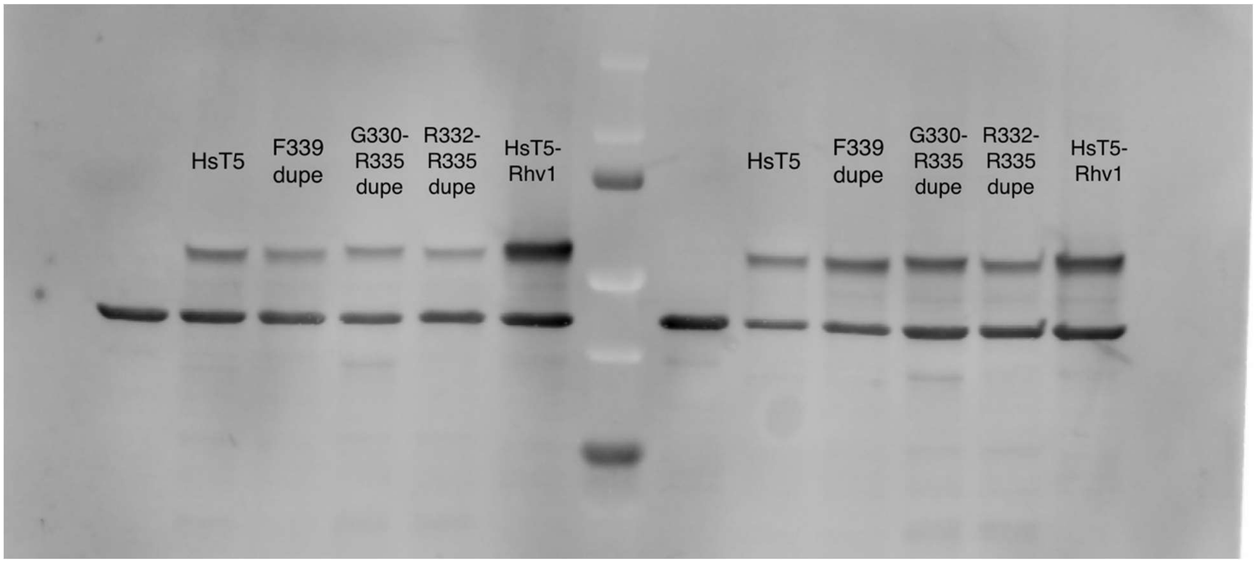

Ran same samples without re-running Bradford. File name: 20240112-WB-14&17-rhv1x2-[IRlong].tif

Figure 4E: immunoblot of HsT5-F339dup mutants.

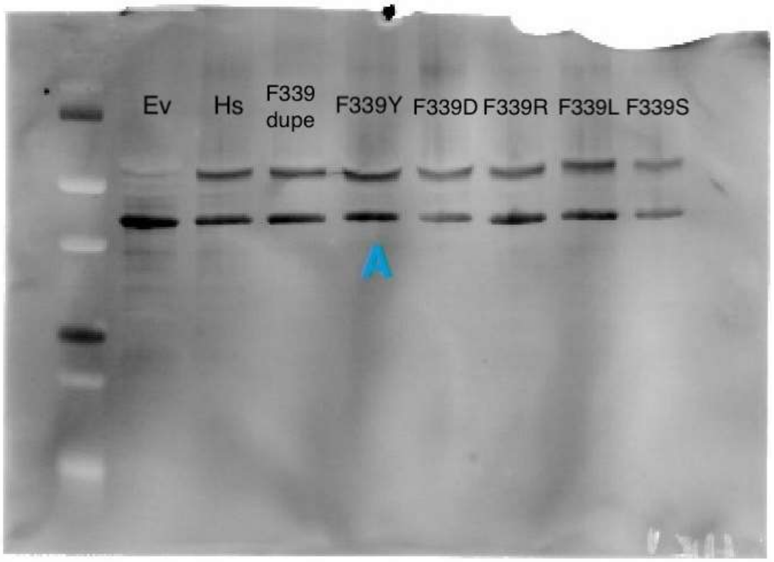

240105-WBf339mut-1215

20240105-WB-F339mut-1218.tif
Sample order: Ev, Hs, F339dupe, F339Y, F339D, F339R, F339L, F339S,

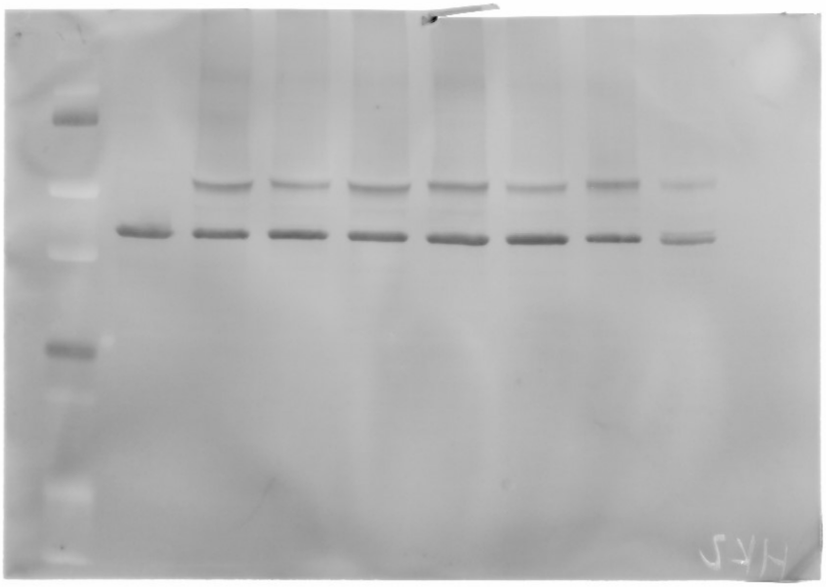

Figure 5B: immunoblot of RhT5 +/- 2aa

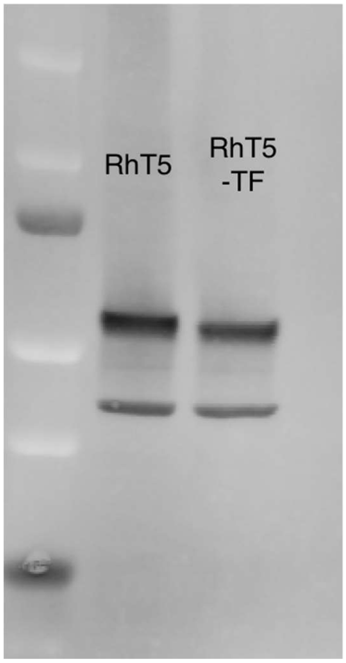

File Name: 20240122-WB-105-etc-[IRlong].tif (Bands after the Third ladder, farthest right bands)

Figure 5C-D: immunoblot of T5 +/- 20aa

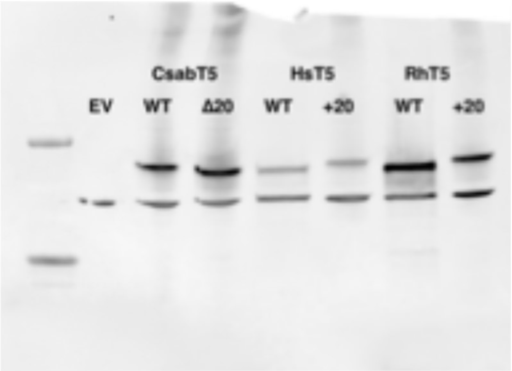

20240125-ir-WB-ET-rep1-[IRlong].tif

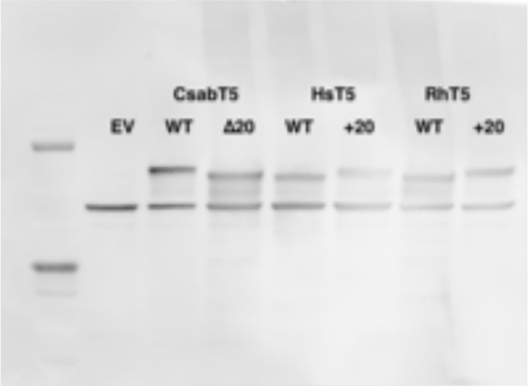

20240130-153228 IR WB-[IRlong].tif – rep 1

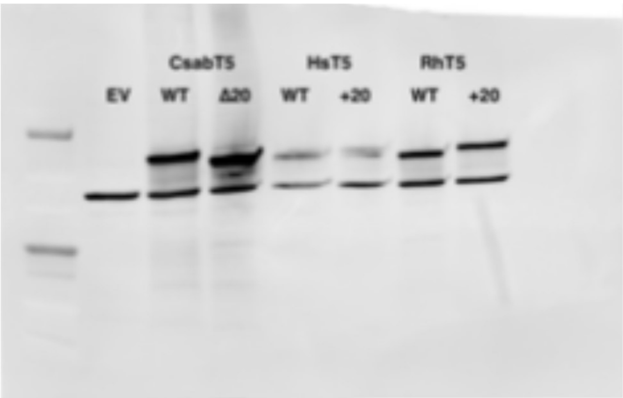

20240130-153228 IR WB-[IRlong].tif – rep 2

Figure S3D: immunoblot of RhT5 to human TRIM5 variants

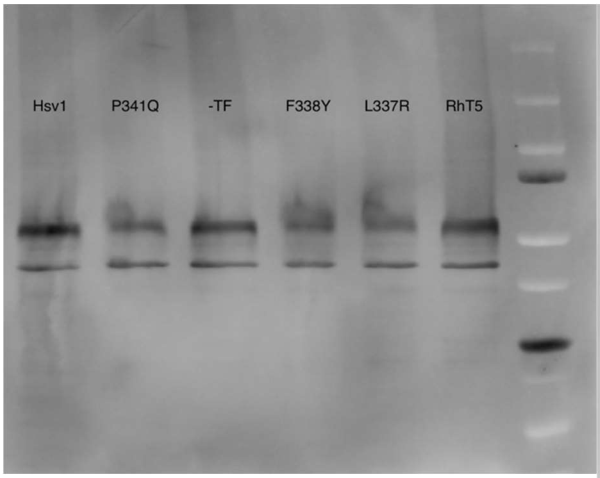

File name: 20240122-WB-Rh20etc-[IRlong].tif

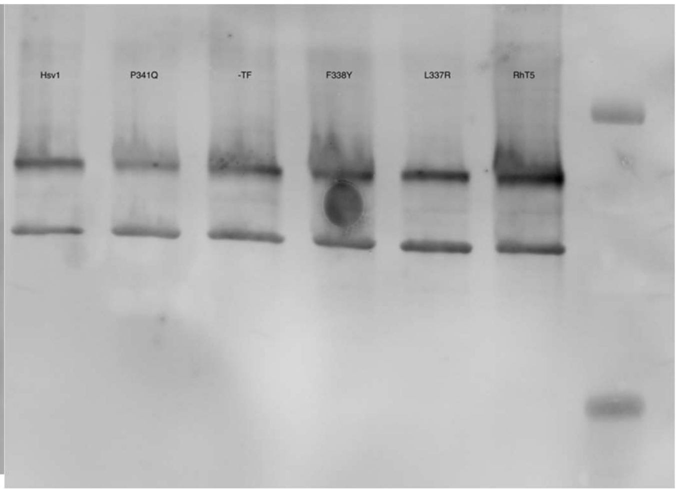

File Name: 20240118-WB-rh&20-[IRlong].tif

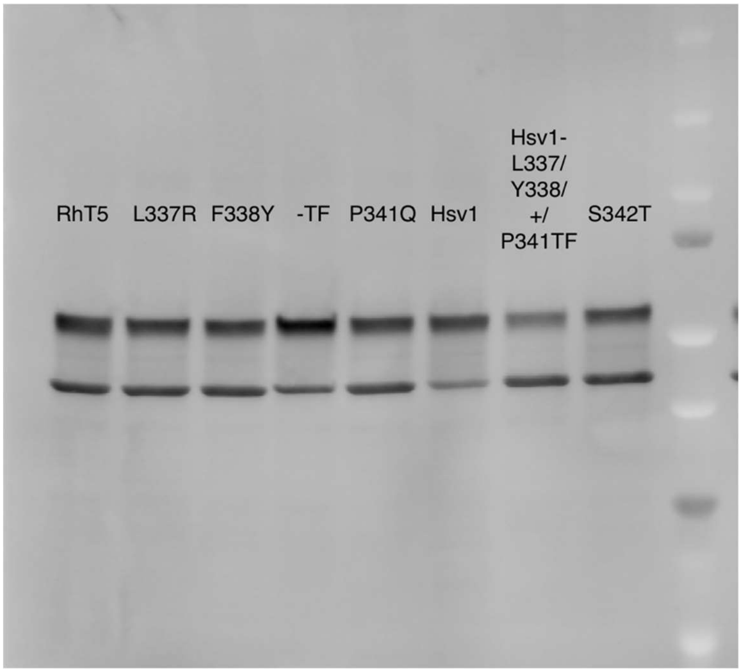

File name: 20240126-WB-Rh20&97-99-[IRlong].tif

Figure S4A: immunoblot of HsT5 +/- TF (RhT5 insertion)

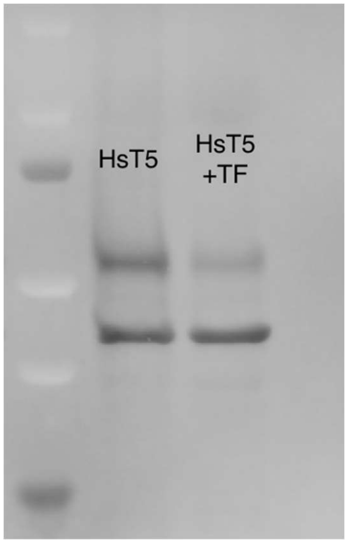

File Name: 20240124-WB-Rh-105etc-[IRlong].tif

Figure S4B: Immunoblot of HsT5-F339dup variants

File name: 20240110-WB-97-99-[IRlong].tif

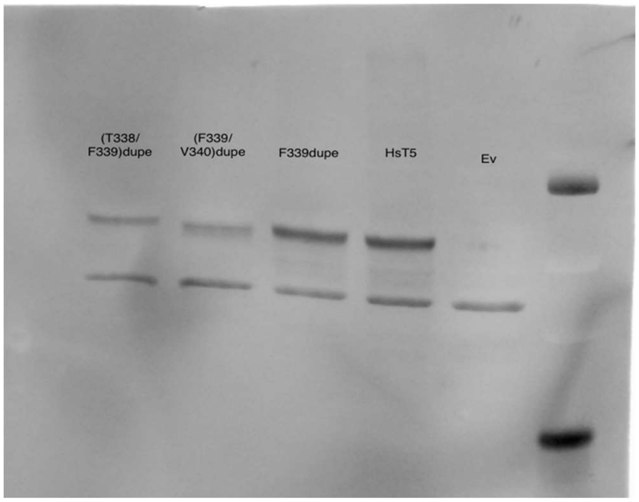

File name: 20240110-WB-97-99-[IRlong].tif

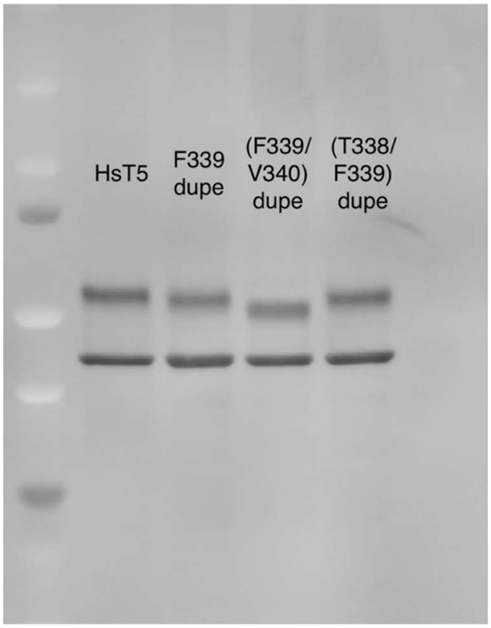

File name: 20240126-WB-Rh20&97-99-[IRlong].tif
